# Supplementary material for: Variable rates of SARS-CoV-2 evolution in chronic infections
Source: PLoS Pathog. 2025 Apr 28;21(4):e1013109. doi: 10.1371/journal.ppat.1013109 (PMC12061394; doi:10.1371/journal.ppat.1013109)
Supplement: S1 Fig — For each patient not so described in the main text, graphical representations of sequence data are shown. Dots are labelled with the day of the collection of the sample they represent and are coloured by the subpopulation with which they were identified by our method. A phylogenetic representation of the sequence data is shown below each of the respective plots. (PDF) [file ppat.1013109.s001.pdf]

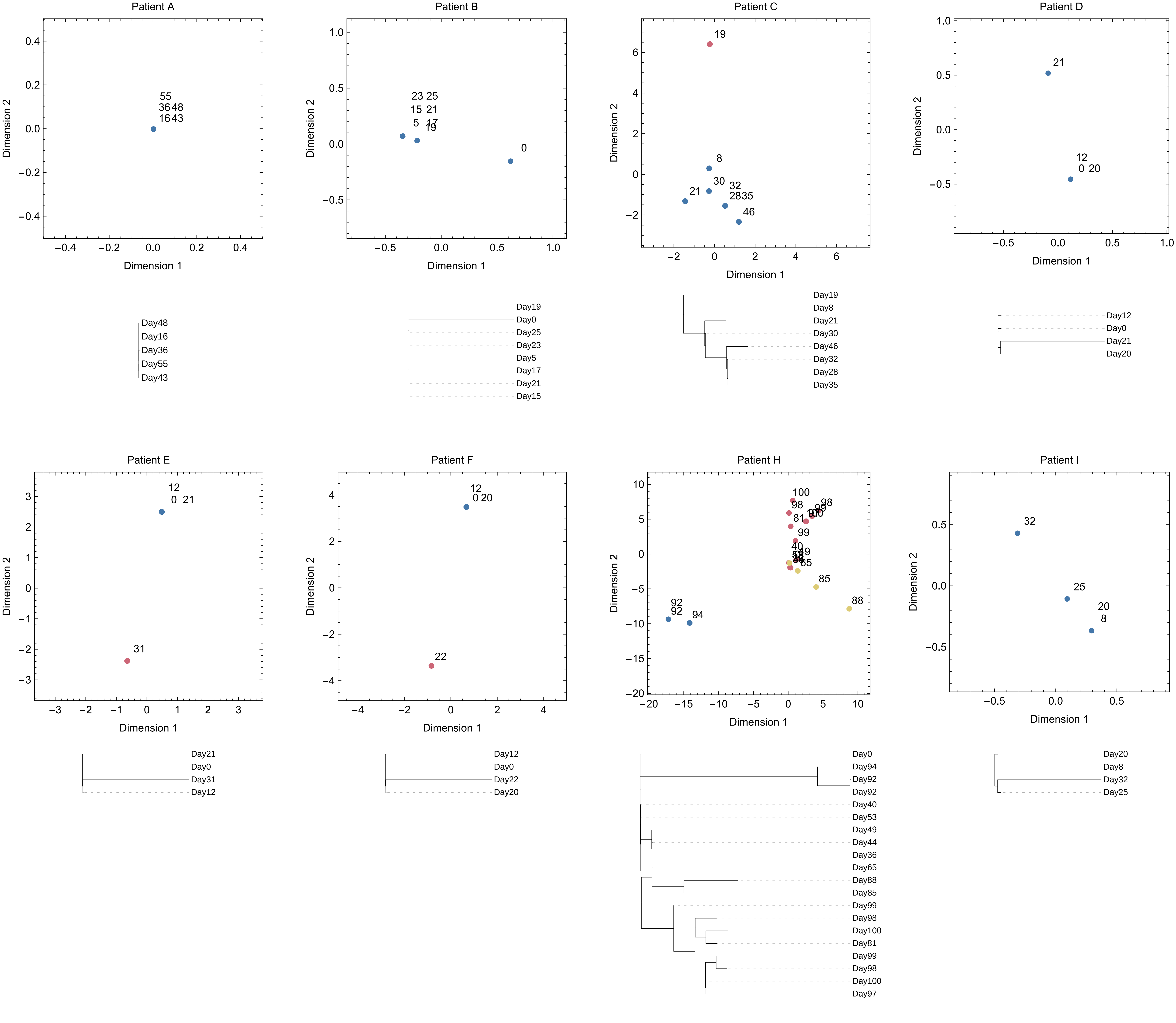

Patient A
Dimension 1
Dimension 2
55
36 48
16 43
Day48
Day16
Day36
Day55
Day43
Patient B
Dimension 1
Dimension 2
23 25
15 21
5 17
19
0
Day19
Day0
Day25
Day23
Day5
Day17
Day21
Day15
Patient C
Dimension 1
Dimension 2
19
8
30
32
21
28 35
46
Day19
Day8
Day21
Day30
Day46
Day32
Day28
Day35
Patient D
Dimension 1
Dimension 2
21
12
0 20
Day12
Day0
Day21
Day20
Patient E
Dimension 1
Dimension 2
12
0 21
31
Day21
Day0
Day31
Day12
Patient F
Dimension 1
Dimension 2
12
0 20
22
Day12
Day0
Day22
Day20
Patient H
Dimension 1
Dimension 2
100
98
98
81
99
40
65
85
88
92
92
94
Day0
Day94
Day92
Day92
Day40
Day53
Day49
Day44
Day36
Day65
Day88
Day85
Day99
Day98
Day100
Day81
Day99
Day98
Day100
Day97
Patient I
Dimension 1
Dimension 2
32
25
20
8
Day20
Day8
Day32
Day25
