# Supplementary figures and images for: Variable rates of SARS-CoV-2 evolution in chronic infections
Source: PLoS Pathog. 2025 Apr 28;21(4):e1013109. doi: 10.1371/journal.ppat.1013109 (PMC12061394; doi:10.1371/journal.ppat.1013109)

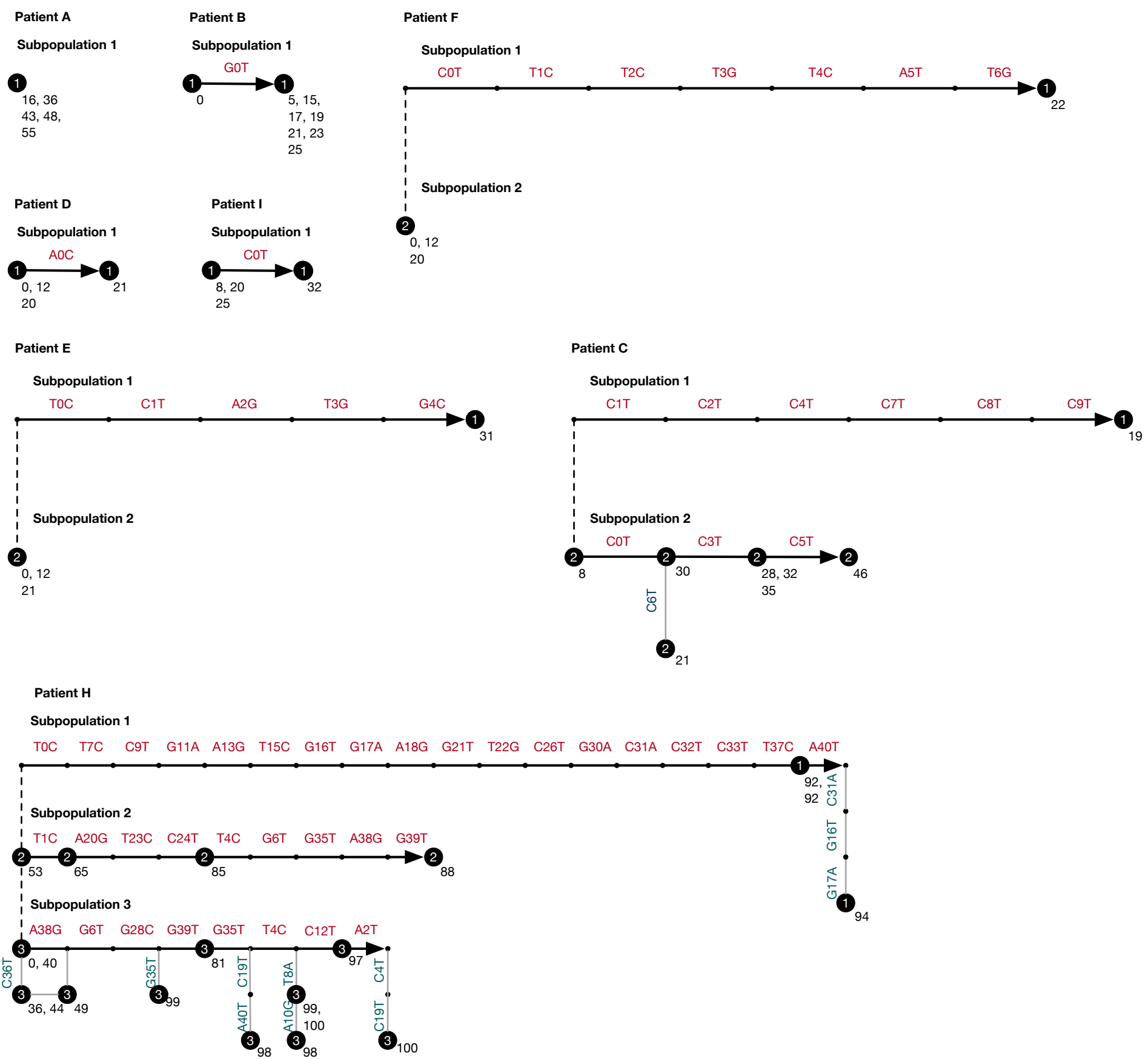

Supplement: S2 Fig — Representations of data show the samples attributed to each population (black, numbered by subpopulation, positioned by sequence). Nucleotide substitutions for each population are indexed from zero. Fixations within subpopulations are shown in red text, while fluctuations are shown in blue text. Maximum likelihood reconstructions are shown; there are often multiple plausible reconstructions of the data. (PDF) [file ppat.1013109.s002.pdf]

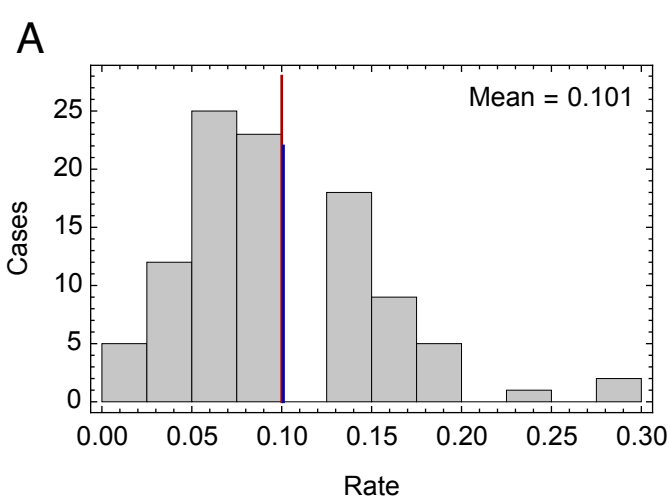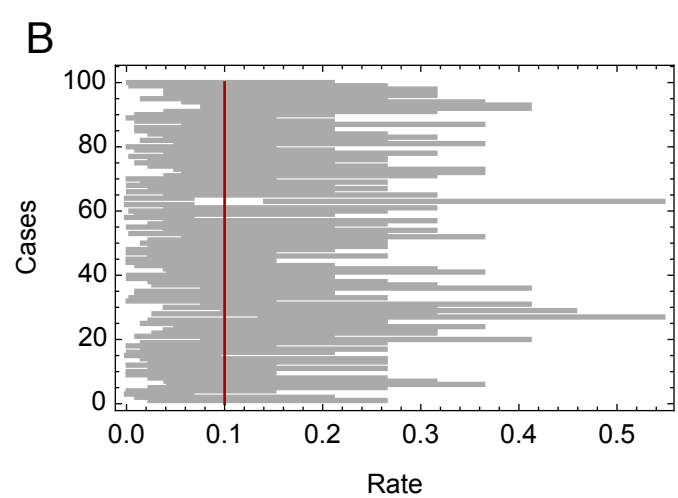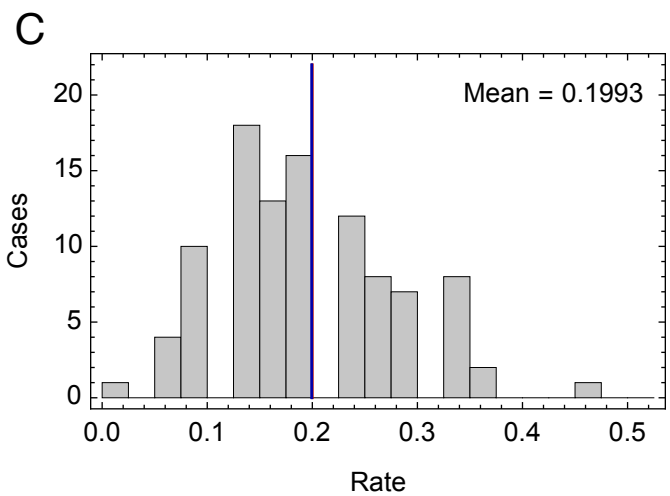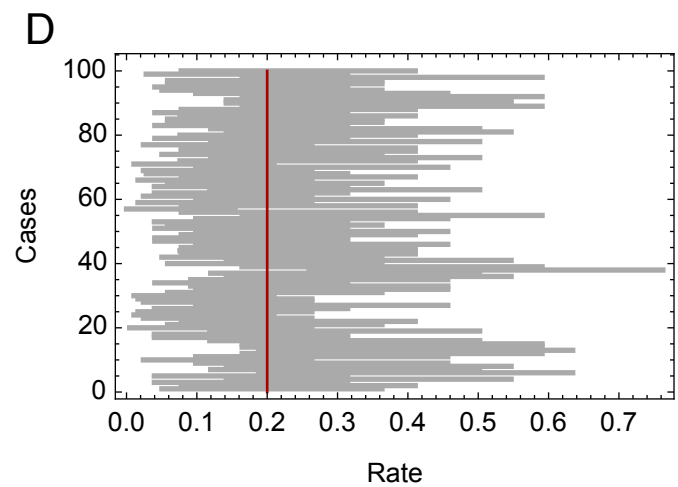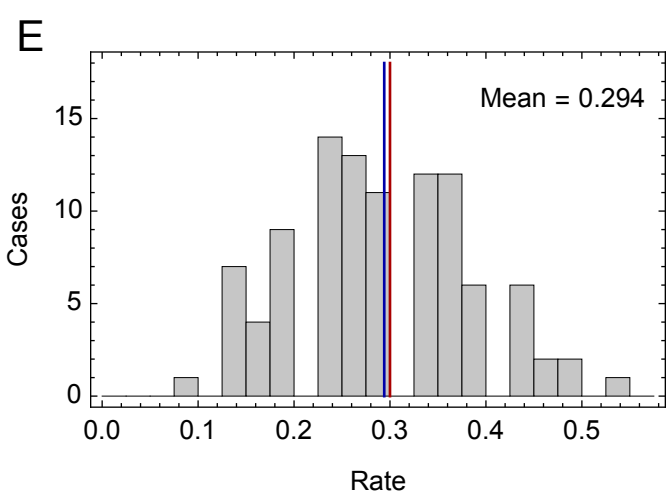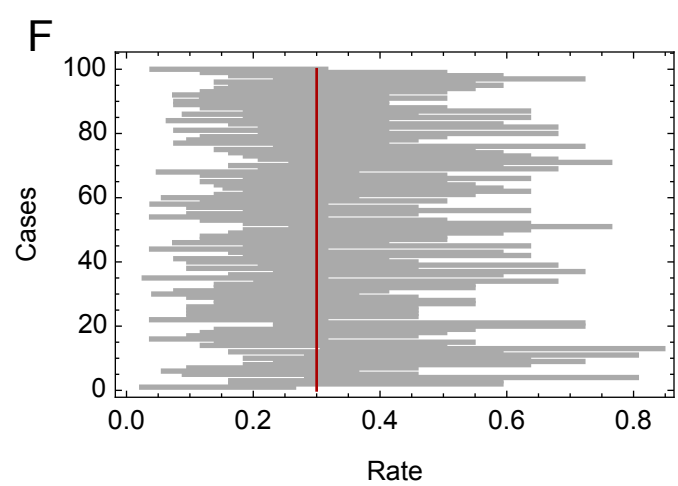

Supplement: S3 Fig — Simulations described simple populations with an underlying rate of evolution, simulated for 30 days. The actual number of mutations gained by a population during this period is Poisson distributed according to the product of the rate and time. A. Inferred rates of evolution (gray bars) given a rate of 0.1 per day. The mean inferred rate across 100 populations (vertical blue line) is very similar to the actual rate (vertical red line). B. Inferred 95% confidence intervals for these inferences. The correct rate of evolution (vertical red line) was contained within 93 of 100 intervals. C. Inferred rates of evolution given a rate of 0.2 per day. The mean inferred rate (vertical blue line) is very similar to the actual rate (vertical red line). D. Inferred 95% confidence intervals for these inferences. The correct rate of evolution (vertical red line) was contained within 98 of 100 intervals. E. Inferred rates of evolution given a rate of 0.3 per day (gray bars). The mean inferred rate (vertical blue line) is very similar to the actual rate (vertical red line). F. Inferred 95% confidence intervals for these inferences. The correct rate of evolution (vertical red line) was contained within 98 of 100 intervals. (PDF) [file ppat.1013109.s003.pdf]

**A**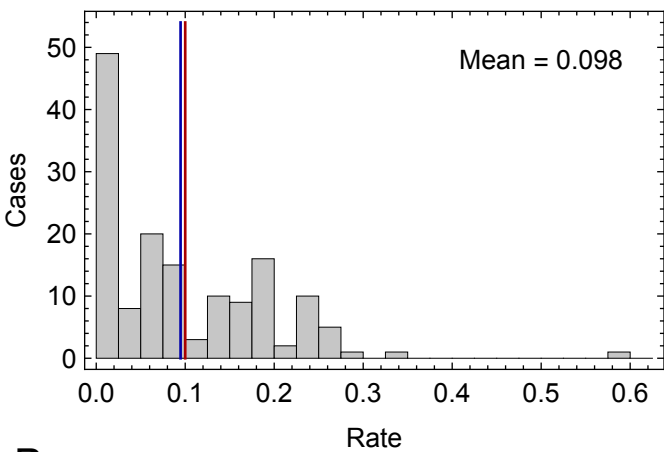**B**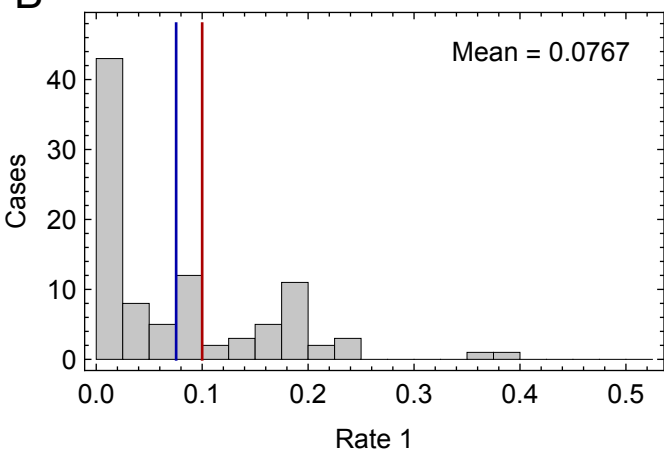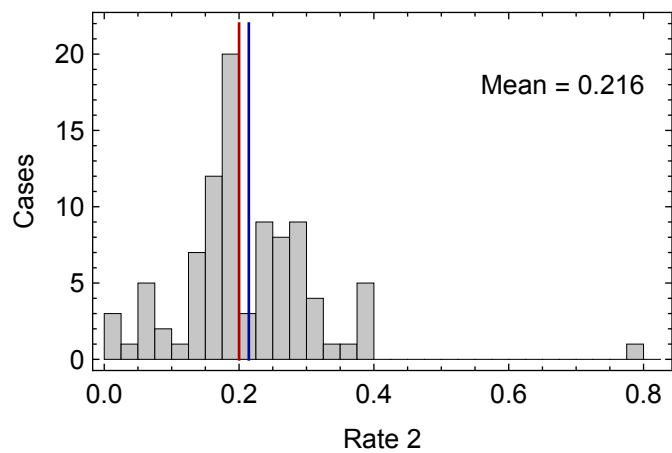**C**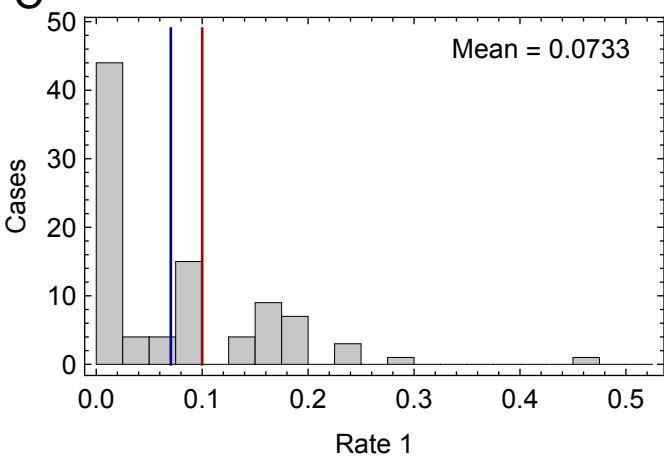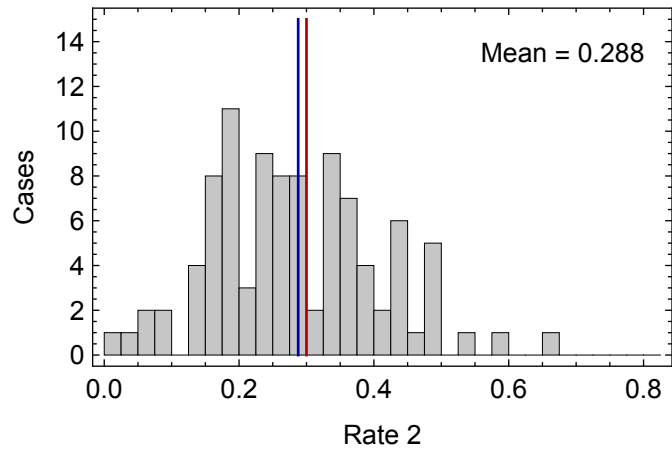

Supplement: S4 Fig — Data describe populations with two subpopulations, simulated for 30 days. A. Inferred rates of evolution (gray bars) where viral populations evolve at rate 0.1. The mean inferred rate (vertical blue line) and actual rate (vertical red line) are shown. B. Inferred rates of evolution (gray bars) where viral populations evolve at rates 0.1 and 0.2. The mean inferred rate (vertical blue line) and actual rate (vertical red line) for each population are shown. C. Inferred rates of evolution (gray bars) where viral populations evolve at rates 0.1 and 0.3. The mean inferred rate (vertical blue line) and actual rate (vertical red line) for each population are shown. (PDF) [file ppat.1013109.s004.pdf]

**A**

Three populations: Rates 0.1, 0.2, 0.3

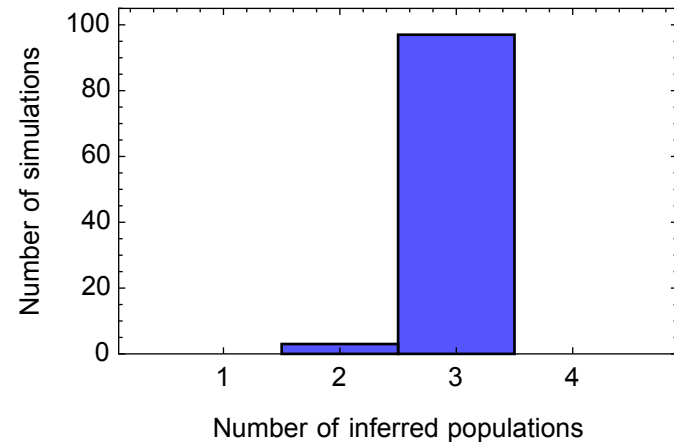**B**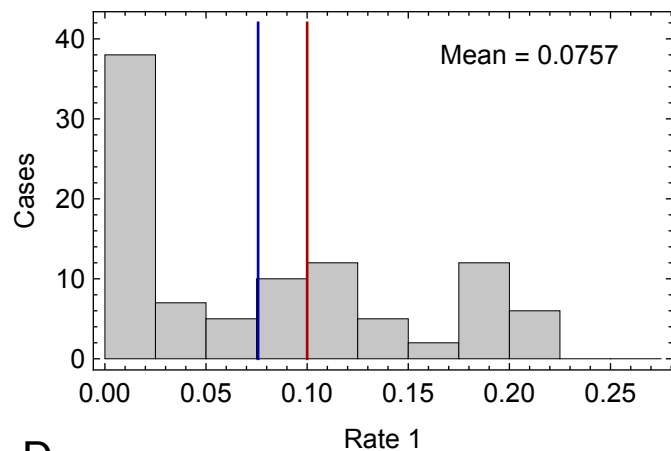**C**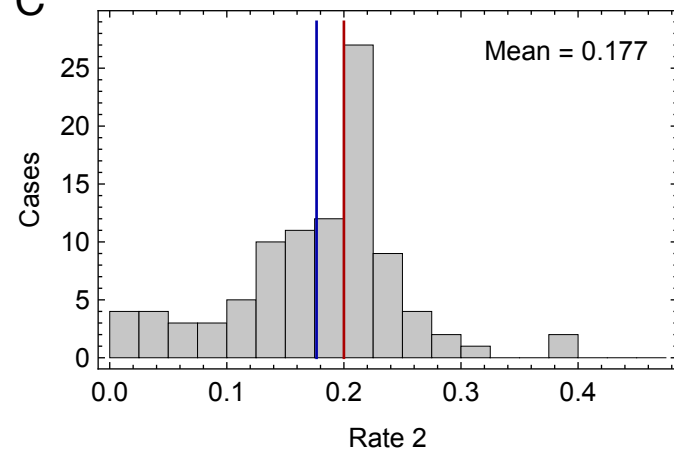**D**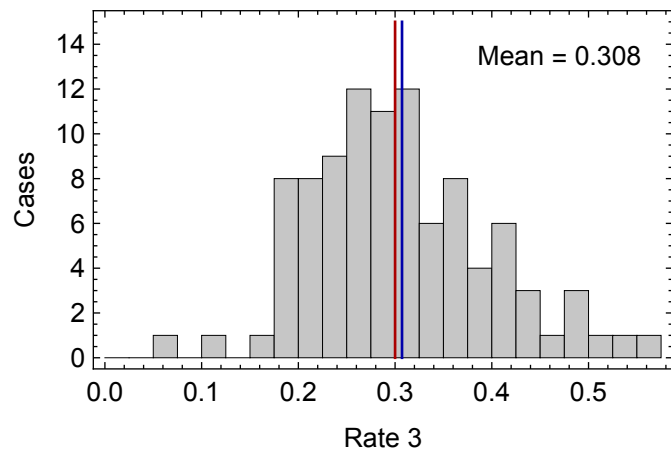

Supplement: S6 Fig — A. Number of subpopulations identified by the model. Three subpopulations were identified in 97 out of 100 simulations. B. Smallest fitness parameter inferred by the model shown as a histogram (gray bars). The red line and blue line show the simulated rate and the mean inferred rate of evolution respectively. C. Intermediate fitness parameter inferred by the model shown as a histogram (gray bars). The red line and blue line show the simulated rate and the mean inferred rate of evolution respectively. D. Largest fitness parameter inferred by the model shown as a histogram (gray bars). The red line and blue line show the simulated rate and the mean inferred rate of evolution. (PDF) [file ppat.1013109.s006.pdf]

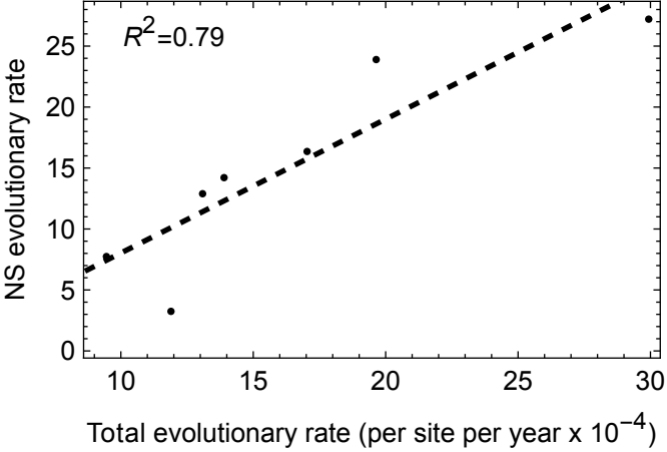

Supplement: S7 Fig — The dashed black line shows a linear model fit to the data. (PDF) [file ppat.1013109.s007.pdf]

Patient A

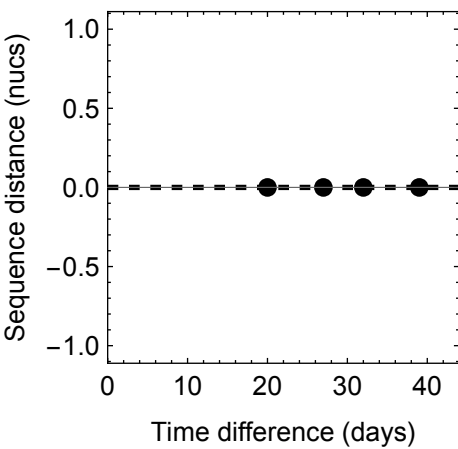

Patient B

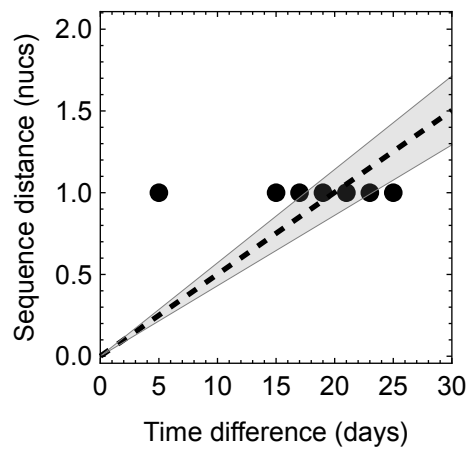

Patient C

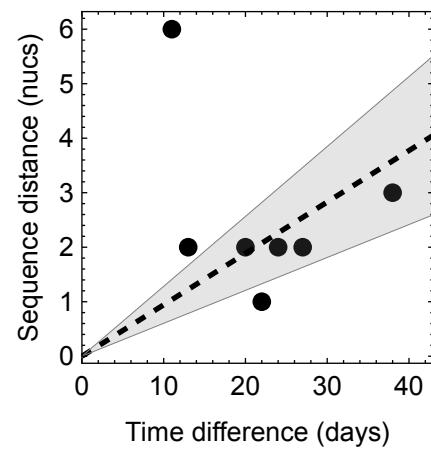

Patient D

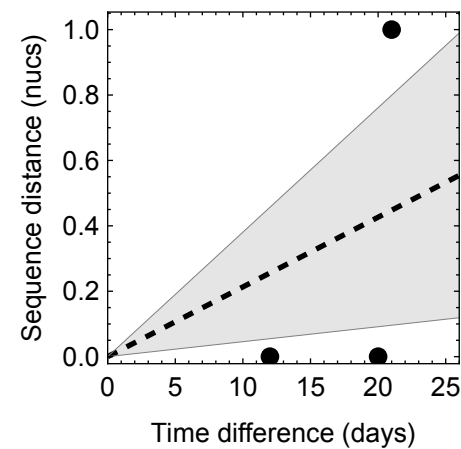

Patient E

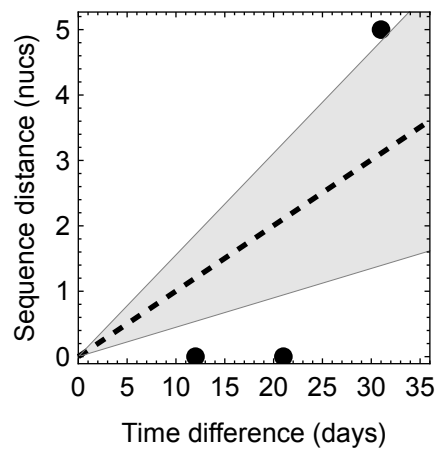

Patient F

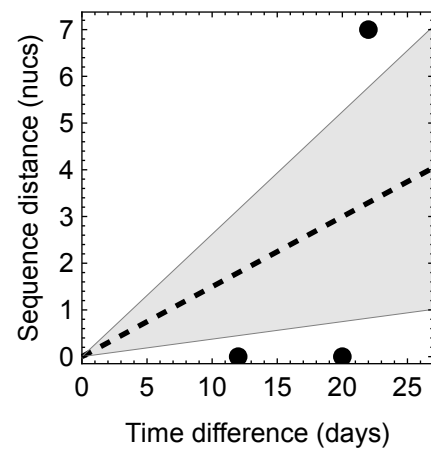

Patient G

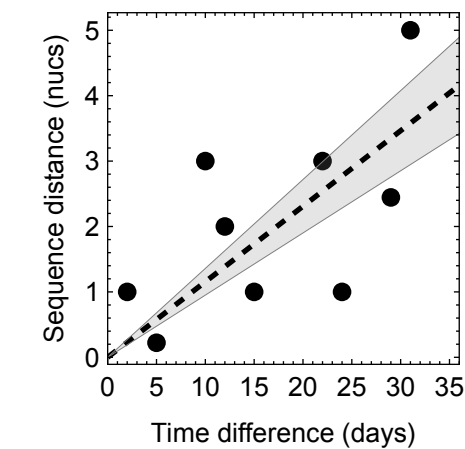

Patient H

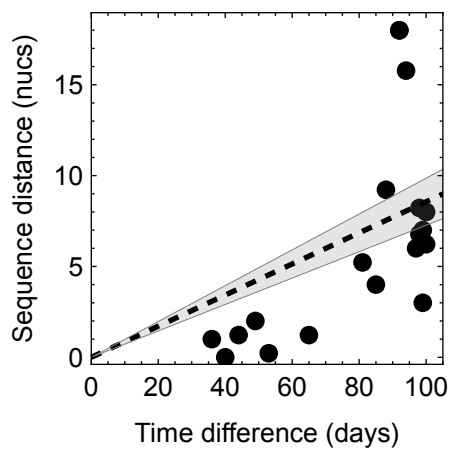

Patient I

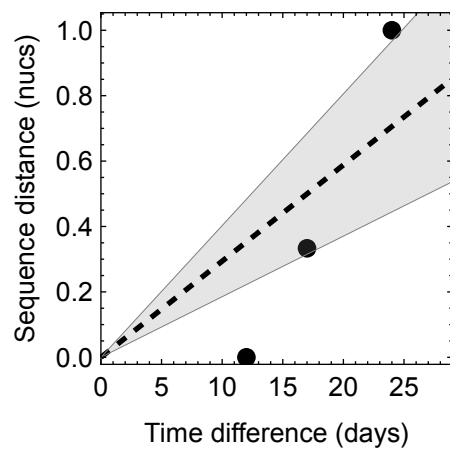

Supplement: S8 Fig — Individual sequences are shown as dots. Non-integer sequence distances were sometimes identified in the case of missing nucleotide data. The black dashed line for each case shows a linear regression model fitted to the data. The gray shaded region shows confidence intervals for this model. (PDF) [file ppat.1013109.s008.pdf]

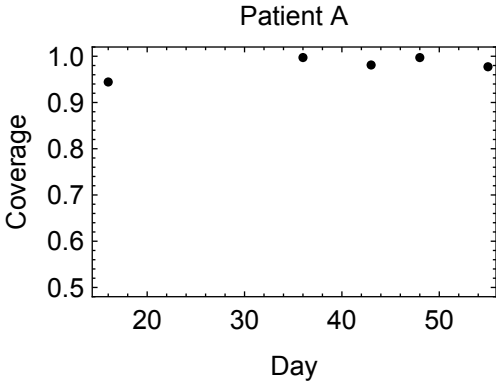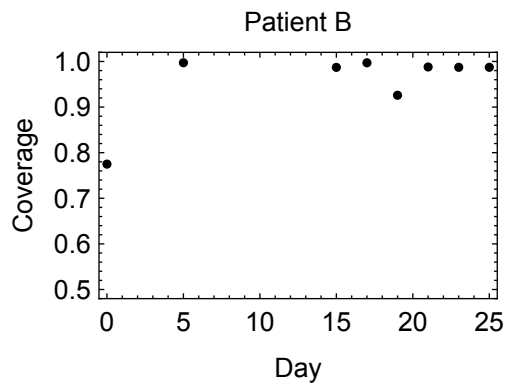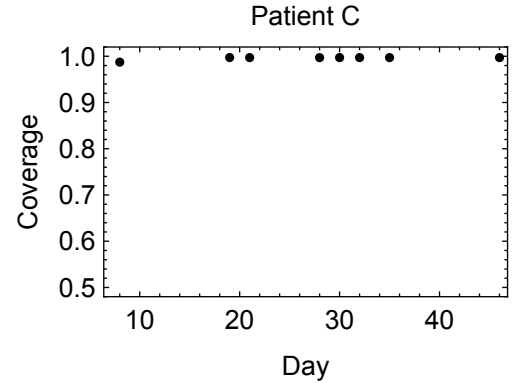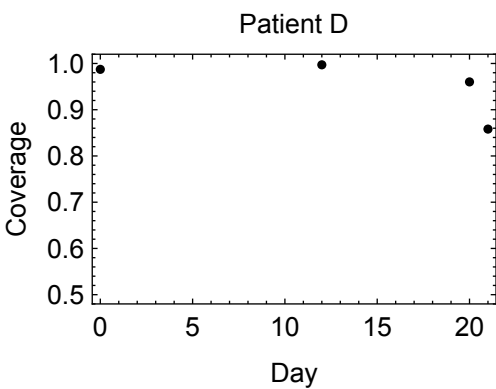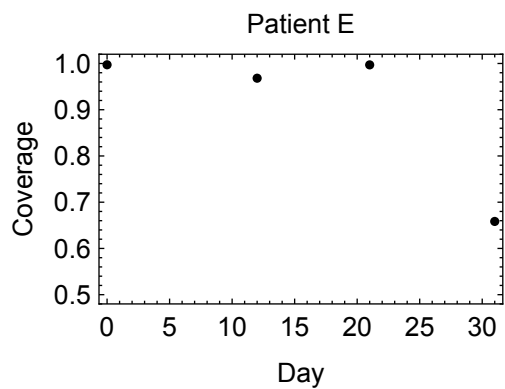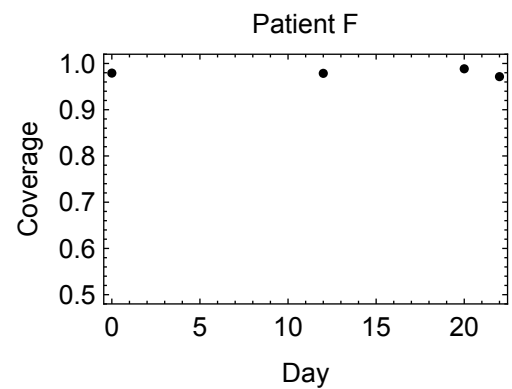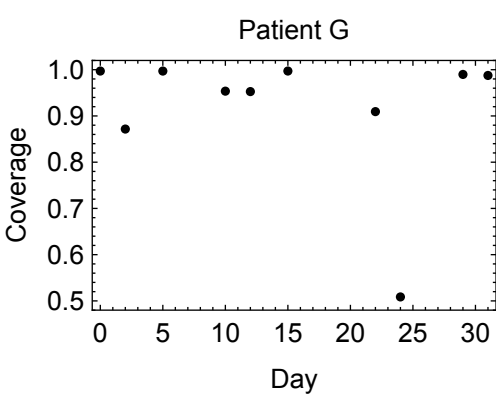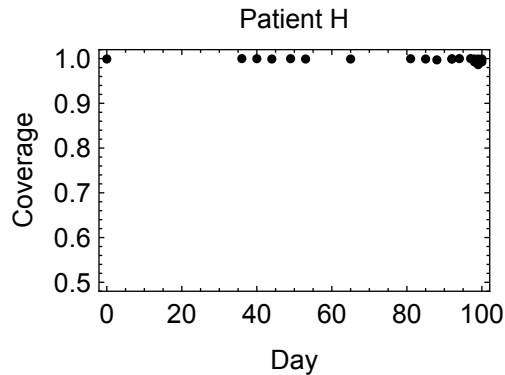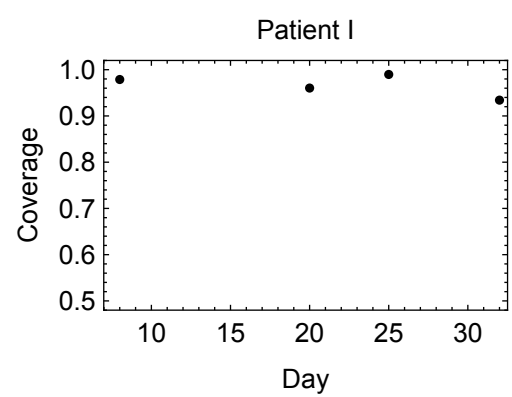

Supplement: S9 Fig — Coverage is described as the fraction of the genome for which unambiguous nucleotides were observed. (PDF) [file ppat.1013109.s009.pdf]

Patient A

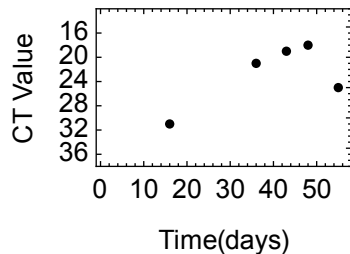

Patient B

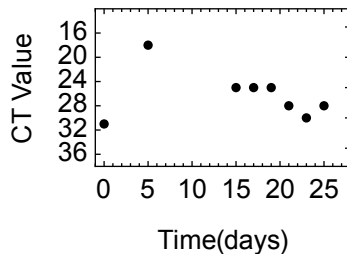

Patient C

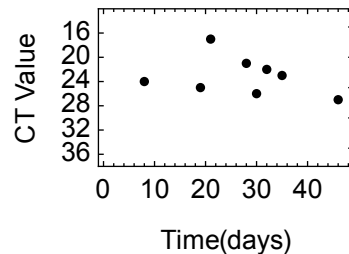

Patient D

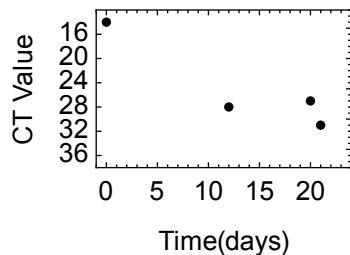

Patient E

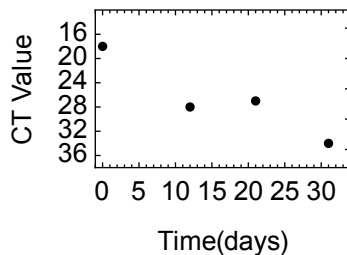

Patient F

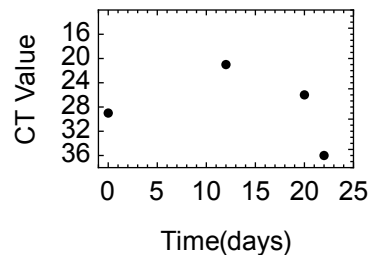

Patient G

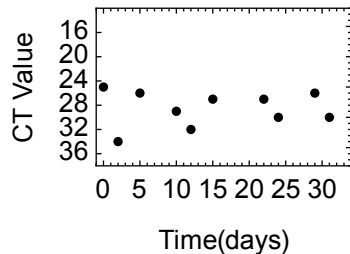

Patient H

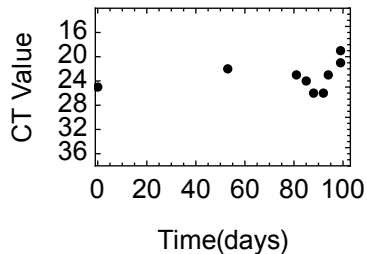

Patient I

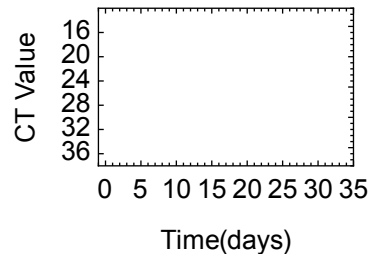

Supplement: S10 Fig — Times are shown relative to the time of symptom onset, where known, or to the time of the first collected sample. Samples from Patient I were generated using the Panther platform, such that no CT values were recorded. (PDF) [file ppat.1013109.s010.pdf]

**One subpopulation**

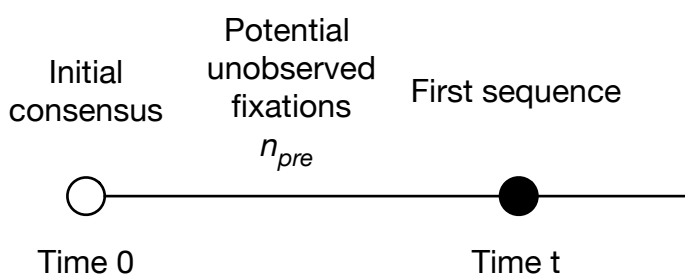

**Two subpopulations**

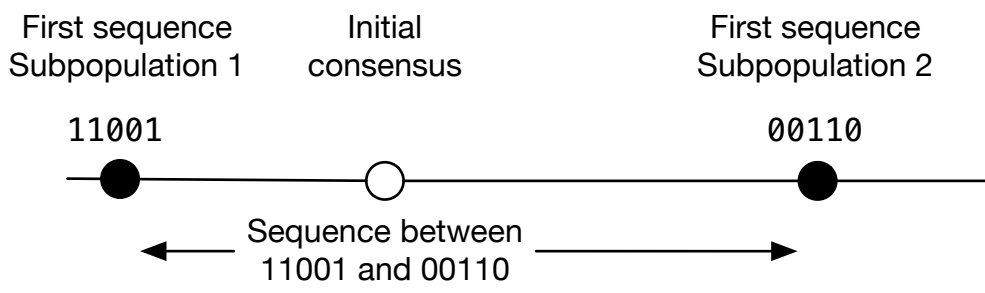

**Three+ subpopulations**

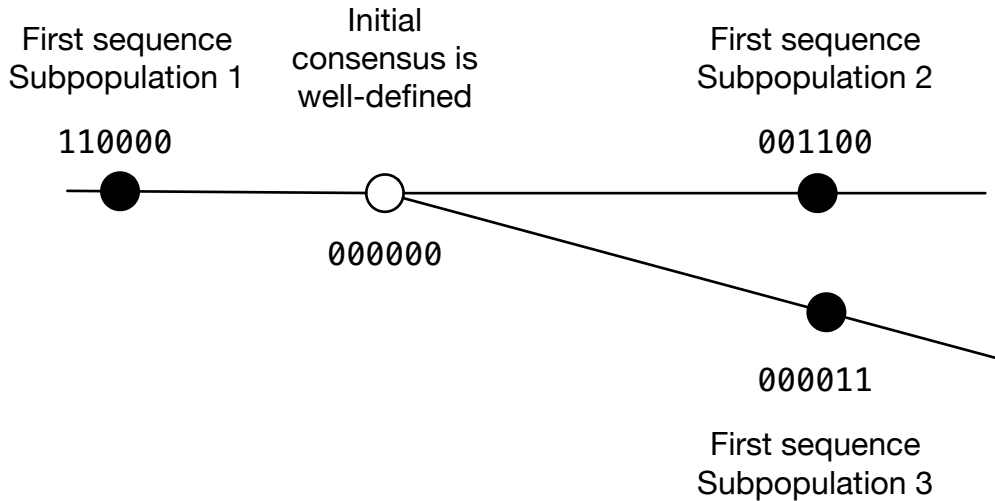

Supplement: S12 Fig — Consensus sequences were defined in a flexible manner, under the assumption that fixations, once gained, are not lost. In model I, with one subpopulation, where the first sample is collected at time t > 0, the potential exists for fixations to have occurred in the population prior to time t. Our model allows for some number npre fixations to have occurred. In model V, with two subpopulations, where the first observed sequences in each subpopulation differ, we allow the consensus to have been either of these two sequences, or any sequence in between them. In models Y and above, with three+ subpopulations, the initial consensus is defined uniquely as not containing any of the variants observed in the first sequences of the distinct populations. Where multiple consensus sequences were possible, the maximum likelihood was calculated across all possible such sequences. (PDF) [file ppat.1013109.s012.pdf]
