# Supplementary material for: Variable rates of SARS-CoV-2 evolution in chronic infections
Source: PLoS Pathog. 2025 Apr 28;21(4):e1013109. doi: 10.1371/journal.ppat.1013109 (PMC12061394; doi:10.1371/journal.ppat.1013109)
Supplement: S5 Fig — Data describe populations with one or two subpopulations, simulated for 30 days. Under-calling of distinct populations occurs in up to 25% of inferences for the case where the rates of evolution of the populations are identical, but over-calling of distinct populations was rare. (PDF) [file ppat.1013109.s005.pdf]

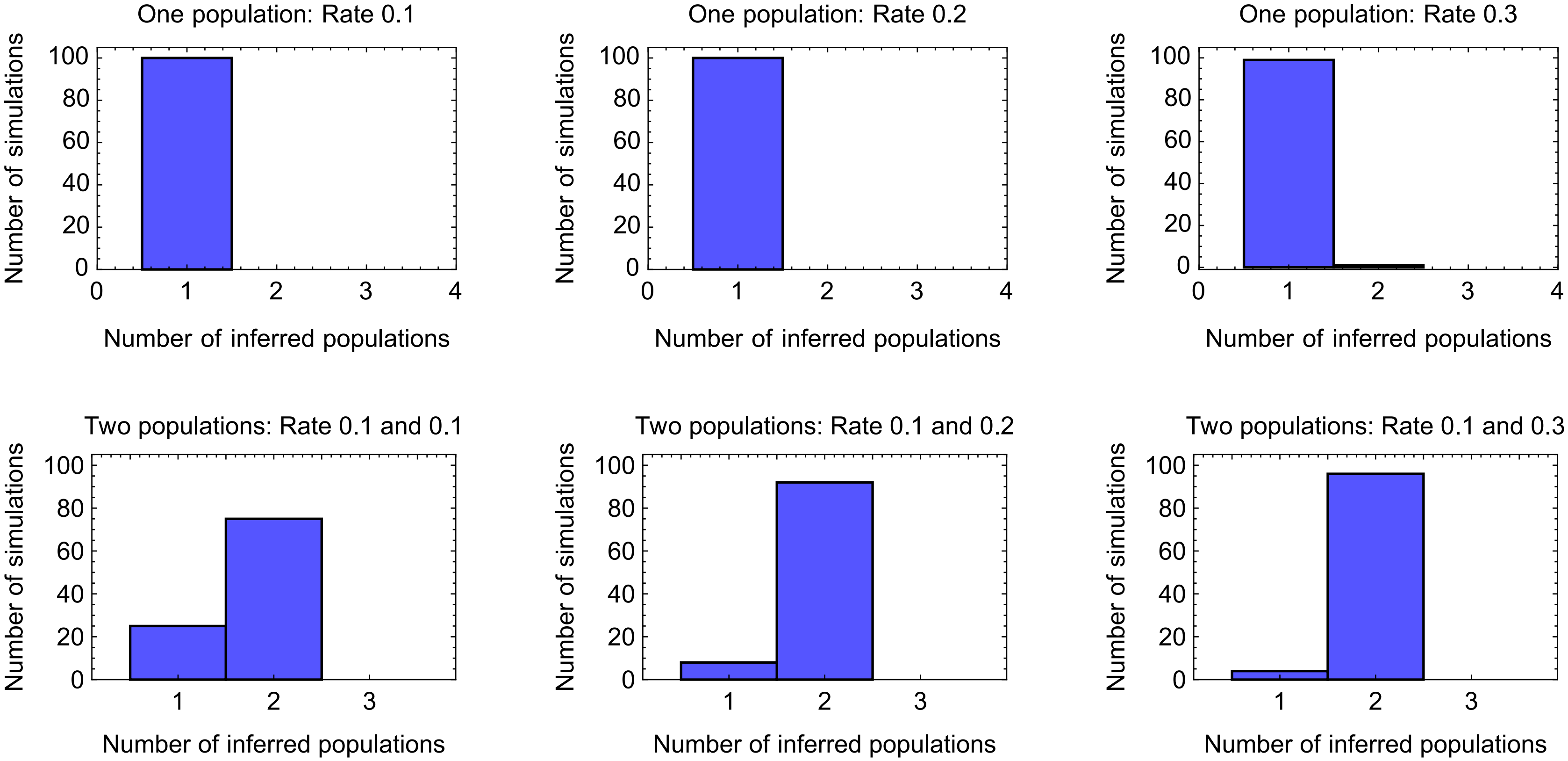

One population: Rate 0.1
One population: Rate 0.2
One population: Rate 0.3
Two populations: Rate 0.1 and 0.1
Two populations: Rate 0.1 and 0.2
Two populations: Rate 0.1 and 0.3
Number of simulations
Number of inferred populations
0
20
40
60
80
100
0
1
2
3
4
