# Supplementary material for: Variable rates of SARS-CoV-2 evolution in chronic infections
Source: PLoS Pathog. 2025 Apr 28;21(4):e1013109. doi: 10.1371/journal.ppat.1013109 (PMC12061394; doi:10.1371/journal.ppat.1013109)
Supplement: S11 Fig — These sets of vectors are used in the separation of fixation and fluctuation events. V0 represents the continual presence or absence of a variant in the population, while V1 represents the fixation of a variant at some point in time. (PDF) [file ppat.1013109.s011.pdf]

Sequence					V0		V1						
0	0	0	0	0	1	0	0	0	0	0	0	0	0
N	0	0	0	0	1	0	1	0	0	0	0	0	0
1	N	0	1	0	1	0	1	1	0	0	0	0	0
1	0	0	0	0	1	0	1	1	1	0	0	0	0
1	1	N	1	0	1	0	1	1	1	1	1	0	0
1	1	0	N	N	1	0	1	1	1	1	1	1	0
1	1	1	1	1	1	0	1	1	1	1	1	1	1
