# Supplementary material for: Variable rates of SARS-CoV-2 evolution in chronic infections
Source: PLoS Pathog. 2025 Apr 28;21(4):e1013109. doi: 10.1371/journal.ppat.1013109 (PMC12061394; doi:10.1371/journal.ppat.1013109)
Supplement: S1 Text — (DOCX) [file ppat.1013109.s016.docx]

**Membership of the ISARIC Consortium**

J Kenneth Baillie,

Malcolm G Semple,

Peter JM Openshaw,

Gail Carson,

Beatrice Alex,

Benjamin Bach,

Wendy S Barclay,

Debby Bogaert,

Meera Chand,

Graham S Cooke,

Annemarie B Docherty,

Jake Dunning,

Ana da Silva Filipe,

Tom Fletcher,

Christopher A Green,

Ewen M Harrison,

Julian A Hiscox,

Antonia Ying Wai Ho,

Peter W Horby,

Samreen Ijaz,

Saye Khoo,

Paul Klenerman,

Andrew Law,

Wei Shen Lim,

Alexander J Mentzer,

Laura Merson,

Alison M Meynert,

Mahdad Noursadeghi,

Shona C Moore,

Massimo Palmarini,

William A Paxton,

Georgios Pollakis,

Nicholas Price,

Andrew Rambaut,

David L Robertson,

Clark D Russell,

Vanessa Sancho-Shimizu,

Janet T Scott,

Thushan de Silva,

Louise Sigfrid,

Tom Solomon,

Shiranee Sriskandan,

David Stuart,

Charlotte Summers,

Richard S Tedder,

Emma C Thomson,

AA Roger Thompson,

Ryan S Thwaites,

Lance CW Turtle,

Maria Zambon,

Hayley Hardwick,

Chloe Donohue,

Ruth Lyons,

Fiona Griffiths,

Wilna Oosthuyzen,

Lisa Norman,

Riinu Pius,

Tom M Drake,

Cameron J Fairfield,

Stephen Knight,

Kenneth A Mclean,

Derek Murphy,

Catherine A Shaw,

Jo Dalton,

James Lee,

Daniel Plotkin,

Michelle Girvan,

Scott Mullaney,

Claire Petersen,

Egle Saviciute,

Stephanie Roberts,

Janet Harrison,

Laura Marsh,

Marie Connor,

Sophie Halpin,

Clare Jackson,

Carrol Gamble,

Gary Leeming,

Andrew Law,

Murray Wham,

Sara Clohisey,

Ross Hendry,

James Scott-Brown,

William Greenhalf,

Victoria Shaw,

Sarah McDonald,

Seán Keating,

Katie A. Ahmed,

Jane A Armstrong,

Milton Ashworth,

Innocent G Asiimwe,

Siddharth Bakshi,

Samantha L Barlow,

Laura Booth,

Benjamin Brennan,

Katie Bullock,

Benjamin WA Catterall,

Jordan J Clark,

Emily A Clarke,

Sarah Cole,

Louise Cooper,

Helen Cox,

Christopher Davis,

Oslem Dincarslan,

Chris Dunn,

Philip Dyer,

Angela Elliott,

Anthony Evans,

Lorna Finch,

Lewis WS Fisher,

Terry Foster,

Isabel Garcia-Dorival,

Willliam Greenhalf,

Philip Gunning,

Catherine Hartley,

Antonia Ho,

Rebecca L Jensen,

Christopher B Jones,

Trevor R Jones,

Shadia Khandaker,

Katharine King,

Robyn T. Kiy,

Chrysa Koukorava,

Annette Lake,

Suzannah Lant,

Diane Latawiec,

L Lavelle-Langham,

Daniella Lefteri,

Lauren Lett,

Lucia A Livoti,

Maria Mancini,

Sarah McDonald,

Laurence McEvoy,

John McLauchlan,

Soeren Metelmann,

Nahida S Miah,

Joanna Middleton,

Joyce Mitchell,

Shona C Moore,

Ellen G Murphy,

Rebekah Penrice-Randal,

Jack Pilgrim,

Tessa Prince,

Will Reynolds,

P. Matthew Ridley,

Debby Sales,

Victoria E Shaw,

Rebecca K Shears,

Benjamin Small,

Krishanthi S Subramaniam,

Agnieska Szemiel,

Aislynn Taggart,

Jolanta Tanianis-Hughes,

Jordan Thomas,

Erwan Trochu,

Libby van Tonder,

Eve Wilcock,

J. Eunice Zhang,

Kayode Adeniji,

Daniel Agranoff,

Ken Agwuh,

Dhiraj Ail,

Ana Alegria,

Brian Angus,

Abdul Ashish,

Dougal Atkinson,

Shahedal Bari,

Gavin Barlow,

Stella Barnass,

Nicholas Barrett,

Christopher Bassford,

David Baxter,

Michael Beadsworth,

Jolanta Bernatoniene,

John Berridge,

Nicola Best,

Pieter Bothma,

David Brealey,

Robin Brittain-Long,

Naomi Bulteel,

Tom Burden,

Andrew Burtenshaw,

Vikki Caruth,

David Chadwick,

Duncan Chambler,

Nigel Chee,

Jenny Child,

Srikanth Chukkambotla,

Tom Clark,

Paul Collini,

Catherine Cosgrove,

Jason Cupitt,

Maria-Teresa Cutino-Moguel,

Paul Dark,

Chris Dawson,

Samir Dervisevic,

Phil Donnison,

Sam Douthwaite,

Ingrid DuRand,

Ahilanadan Dushianthan,

Tristan Dyer,

Cariad Evans,

Chi Eziefula,

Chrisopher Fegan,

Adam Finn,

Duncan Fullerton,

Sanjeev Garg,

Sanjeev Garg,

Atul Garg,

Effrossyni Gkrania-Klotsas,

Jo Godden,

Arthur Goldsmith,

Clive Graham,

Elaine Hardy,

Stuart Hartshorn,

Daniel Harvey,

Peter Havalda,

Daniel B Hawcutt,

Maria Hobrok,

Luke Hodgson,

Anil Hormis,

Michael Jacobs,

Susan Jain,

Paul Jennings,

Agilan Kaliappan,

Vidya Kasipandian,

Stephen Kegg,

Michael Kelsey,

Jason Kendall,

Caroline Kerrison,

Ian Kerslake,

Oliver Koch,

Gouri Koduri,

George Koshy,

Shondipon Laha,

Steven Laird,

Susan Larkin,

Tamas Leiner,

Patrick Lillie,

James Limb,

Vanessa Linnett,

Jeff Little,

Michael MacMahon,

Emily MacNaughton,

Ravish Mankregod,

Huw Masson,

Elijah Matovu,

Katherine McCullough,

Ruth McEwen,

Manjula Meda,

Gary Mills,

Jane Minton,

Mariyam Mirfenderesky,

Kavya Mohandas,

Quen Mok,

James Moon,

Elinoor Moore,

Patrick Morgan,

Craig Morris,

Katherine Mortimore,

Samuel Moses,

Mbiye Mpenge,

Rohinton Mulla,

Michael Murphy,

Megan Nagel,

Thapas Nagarajan,

Mark Nelson,

Igor Otahal,

Mark Pais,

Selva Panchatsharam,

Hassan Paraiso,

Brij Patel,

Natalie Pattison,

Justin Pepperell,

Mark Peters,

Mandeep Phull,

Stefania Pintus,

Jagtur Singh Pooni,

Frank Post,

David Price,

Rachel Prout,

Nikolas Rae,

Henrik Reschreiter,

Tim Reynolds,

Neil Richardson,

Mark Roberts,

Devender Roberts,

Alistair Rose,

Guy Rousseau,

Brendan Ryan,

Taranprit Saluja,

Aarti Shah,

Prad Shanmuga,

Anil Sharma,

Anna Shawcross,

Jeremy Sizer,

Manu Shankar-Hari,

Richard Smith,

Catherine Snelson,

Nick Spittle,

Nikki Staines,

Tom Stambach,

Richard Stewart,

Pradeep Subudhi,

Tamas Szakmany,

Kate Tatham,

Jo Thomas,

Chris Thompson,

Robert Thompson,

Ascanio Tridente,

Darell Tupper-Carey,

Mary Twagira,

Andrew Ustianowski,

Nick Vallotton,

Lisa Vincent-Smith,

Shico Visuvanathan,

Alan Vuylsteke,

Sam Waddy,

Rachel Wake,

Andrew Walden,

Ingeborg Welters,

Tony Whitehouse,

Paul Whittaker,

Ashley Whittington,

Meme Wijesinghe,

Martin Williams,

Lawrence Wilson,

Sarah Wilson,

Stephen Winchester,

Martin Wiselka,

Adam Wolverson,

Daniel G Wooton,

Andrew Workman,

Bryan Yates,

Peter Young
