# Supplementary material for: Variable rates of SARS-CoV-2 evolution in chronic infections
Source: PLoS Pathog. 2025 Apr 28;21(4):e1013109. doi: 10.1371/journal.ppat.1013109 (PMC12061394; doi:10.1371/journal.ppat.1013109)
Supplement: Attachment — Submitted filename: Response to reviewers.docx [file ppat.1013109.s018.docx]

We thank the reviewers for their consideration of our manuscript. Comments from the reviewers are included below in black text. Our responses are given in red text, with quotes from the revised manuscript in blue text.

**Part I - Summary**
Please use this section to discuss strengths/weaknesses of study, novelty/significance, general execution and scholarship.

Reviewer #1: This is a very interesting study investigating the within host evolutionary rate of SARS-CoV-2. The authors developed novel methods to estimate such rates and used a very well curated data set for this purpose. The key findings are that the virus often displays population structure within hosts and that it can evolve much faster than estimates at the host-population level.

Overall, I think that this is a solid piece of work and I do not have fundamental concerns. I would like, however, to point out a few things that I deem necessary to improve the clarity. I anticipate that they will require additional work and rewriting, but not additional analyses:

Reviewer #2: This manuscript analyzes sequencing data from chronic SARS-CoV-2 infections and finds evidence in multiple patients for the co-existence of multiple viral lineages, sometimes evolving at different rates. This is an important topic (as the manuscript points out, the Variants of Concern were likely produced by evolution within chronic infections) and this study advances our understanding by characterizing how the virus diversified in multiple infections. So at a high level I found this to be an exciting manuscript. My main concern is that I couldn't understand the central method of the paper for inferring the subpopulation structure, and in the end I'm not even sure how to interpret a subpopulation in this context.

We thank the reviewers for their comments and enthusiasm for our work. Our responses in detail are included below the reviewers, but we first make some preliminary comments.

In the process of working out how better to describe our method, we created some new visualisations of the model output. Doing this for patient G, we felt that the output contained some aspects we didn’t really believe, whereby the maximum likelihood model implied the repeated gain of variants in independent populations. To deal with this we modified our method, adding a statistical penalty against the repeated gain of variants in different populations.

Revalidating the code against simulated data provides almost identical results to our previous code, but some of our results have now changed. Some confidence intervals in our patient data are larger, while data from Patient E now favours a two-population interpretation.

This change in the results leads to a significant association between identifying non-trivial population structure in a host, and the host being severely immunocompromised. We report this association, but are clear in the discussion that there are multiple factors which could be the cause: The association is with the detection of population structure, rather than the existence of population structure.

**Part II – Major Issues: Key Experiments Required for Acceptance**
Please use this section to detail the key new experiments or modifications of existing experiments that should be absolutely required to validate study conclusions.

Generally, there should be no more than 3 such required experiments or major modifications for a "Major Revision" recommendation. If more than 3 experiments are necessary to validate the study conclusions, then you are encouraged to recommend "Reject".

Reviewer #1: - The methods are not detailed enough to assess them properly. I am particularly interested in the inference of subpopulations, but I had to sketch it by hand to grasp what is actually going on. The equations referring to Poisson processes are useful, yet I would encourage the authors to include something graphic to illustrate the initial consensus sequence and how it can evolve into populations or not. This is an important point because this method could potentially become very useful to other researchers if they can follow it.

Provide an illustrated guide to one of the cases.

We have rewritten the methods, hopefully with the result of making them clearer. In Figure 1 we now show an interpretation of the data from Patient G, viewed from the perspective of our method. This is augmented by a new Figure 7, which shows how the sequence data are processed, again giving the example of the data from Patient G. Our method converts sequence data into one or more subpopulations, with each subpopulation being represented by a series of times at which sequences were observed, and the numbers of fixation and fluctuation events observed in each sequence. The paragraph about V_0_ and V_1_ was potentially a sticking point: We have shown the sets of vectors in a new S10 Figure. We also note that the determination of a consensus sequence is not straightforward, requiring a section of the Methods in itself. We have illustrated how this was done in different models via a new S11 Figure.

- One of the github repositories does not work (the link leads to an error) and the other has no documentation, only C++ code. I do not think that this needs to be very extensive, but sufficient guidelines to analyse a toy data set would be immensely useful.

Apologies for this; this was unfortunate. The broken link contained a full stop, which prevented it from accessing the online IVY repository. Instructions for using each piece of code were contained in a file called Instructions.txt within each repository. The IVY repository contains data and scripts allowing for the replication of the calculations in this manuscript, including the generation and analysis of simulation data.

The front-end documentation for both repositories has been improved.

- There needs to be more detail with the phylogenetic tree inference. Because the data involve very low genetic divergence, then the substitution model and likelihood replicates are especially important to determine the reliability of the inferences. I am sure there are no errors here, a few lines with details are needed (substitution model used, etc…).

This section was somewhat neglected; phylogenetic inference was only used for illustrative purposes. We have added more details of the method used for inference, and clarified that this was used only to generate illustrations. Our approach to estimating rates of evolution differs fundamentally from the phylogenetic approach; we made the decision to show phylogenetic trees as they show differences between sequences in a visual and commonly understood manner.

Reviewer #2: 1. There were many details in the description of the method that I could not understand, but at an overarching level, I think it would help to have a clearer description of the biology that the method is trying to capture, say a description of the authors' underlying mental model for the population that drove their methodological choices. I think I can mostly guess it from the setup of the simulations, but not entirely. As an example of how such a biological motivation could help, I think I could follow the algorithm in lines 424-435 as just an abstract series of steps. But it would be much easier to grasp if the manuscript told us what the steps meant biologically.

We have clarified the Methods section of the manuscript with a rewrite and some new Figures.

The algorithm previously in lines 424-435 is a simple characterisation of variants into those which fixed in a population and those which did not, but were transiently observed. We have added a supplementary figure to explain this.

Regarding the biological motivation, we have added a short paragraph to the methods describing the biological assumptions behind the model.

Our inference method is based upon a framework of biological assumptions. Viral infection is founded in a host by a population of genetically similar viruses. Viruses causing infection may comprise either a single coherent population of viruses, or more than one population, with populations being spatially segregated in the respiratory system, such that mixing between segregated populations is negligible, and each population evolves within a host in an independent manner. To the extent that selection acts upon viral populations, parallel adaptation, in the sense of specific mutations fixing in more than one independent populations, is rare. We note that the majority of cases in this study were not treated with specific antiviral therapies, due to their occurrence early in the pandemic. [line 541ff.]

Other assumptions also apply, for example that consensus viral sequence data may contain variants that do not represent the gain of substitutions in an underlying population. This differs from the assumption of a phylogenetic model, which fits a rate of evolution that encompasses all the nucleotide differences observed between sequences. We would argue that our approach is superior in this respect, considering sequences as they truly are, as consensus measurements of a mixed population or populations.

2. I am particularly concerned about the method because it seems to be producing some counterintuitive results. As I understand Figure 5B, it seems to be saying that there is extensive allele sharing across subpopulations, with the major mutant alleles in each subpopulation appearing as minor alleles in the other subpopulations. How are we supposed to interpret this? What do these subpopulations represent? It would seem to be much more natural to think in terms of lineages. Then there would simply be three mutant lineages (D796H / Delta69-70, P330S / W640G, and T240I / Y200H), each present at different frequencies at the different timepoints. The rate of evolution within a lineage is then the rate of substitutions along a line of descent. Since the current subpopulations almost line up with lineages, it seems like this change could be made without much change to the downstream results, but with a huge increase in clarity and interpretability.

This is not a complete understanding of our model, but makes a helpful and valuable point, highlighting the need for additional explanation. The main clarification to be made here is that the short-read data was not used in the identification of subpopulations: Our method works solely on the basis of viral consensus sequences, which were all we had for 8 out of 9 cases.

We have reworded this part of the manuscript appropriately:

Output from our analysis, which was based upon consensus sequence data only, was used to interpret short-read sequence data collected from patient H, described in an earlier study. [line 285ff.]

We note the potential to further develop our method:

The data of Figure 5 highlight limits in our method and potential future avenues for development. For example, the D796H variant, which appears at consensus level in samples assigned to subpopulation 2, appears as a minor variant in samples attributed to subpopulations 1 and 3 (Figure 5B), suggesting that viral material collected in a single sample may not arise solely from one underlying population. A method accounting for the detail of short-read viral sequence data would likely provide more information. [line 319 ff.]

**Part III – Minor Issues: Editorial and Data Presentation Modifications**
Please use this section for editorial suggestions as well as relatively minor modifications of existing data that would enhance clarity.

Reviewer #1: Lines 177 to 179: I found it very interesting that the upper range for the evolutionary rate, of 3.9E-3 subs/site/year, for within host evolution here is comparable to phylogenetic estimates of the precursor lineages of variants of concern (about 2.45E-3 subs/site/year, Tay et al. 2022) using consensus sequences from individual patients. Can the authors comment on whether this may suggest a role in subpopulation structure in the emergence of variants (probably in the Discussion)?

The link to the Tay et al paper is super-relevant; we had not fully considered this. We don’t want to speculate too much but have added to the Discussion as follows

…it is interesting that the highest rates of evolution that we infer within-host (max. likelihood 3.9 x 10^-3^ substitutions per site per year, upper bound 7.9 x 10^-3^) are comparable with those inferred for branches leading to the emergence of novel SARS-CoV-2 variants (Beta 2.8 x 10^-3^ per site per year; Alpha 8.5 x 10^-3^ ^42^). The rapid evolutionary processes generating novel variants may have occurred in compartmentalised populations. [line 491ff.]

L 244: One reason for why regression based estimates might be lower is the presence of pseudoreplication. Conceptually, if evolution is tree like and we assume some degree of shared ancestry between samples, then the deep branches in a tree would be traversed multiple times. These branches tend to have fewer transitory mutations and because they are counted multiple times, they can impose a bias on estimates of evolutionary rates. This is a hypothesis I have worked with, but would be most interested to know what the authors think.

Our thought is that pseudoreplication will increase the variance in the rate of evolution that is inferred by regression, but we are not sure that it would lead to a systematic overestimate or underestimate.

Estimates of evolutionary rate are noisy. When we apply our inference method to simulated data we get a wide range of results when looking at specific instances of evolution; we report the mean inference versus the real rate of evolution as this what gives us confidence that our method does what we think it should. Essentially, evolution is a stochastic process (we assume the number of variants gained is the result of a Poisson process). Each simulation produces an instantiation of that process, leading to an inference of rate that depends on the actual number of mutations which occurred rather than upon the underlying rate.

Now suppose we have data describing evolution from a within-host evolutionary process. If the underlying process of evolution follows a star-shaped phylogeny, whereby every sample is collected from a different and independent population, but each population has the same underlying rate of evolution λ mutations per day. If sample i is collected on day t_i_ of infection, it will have acquired Poisson(λt_i_) mutations. Considering the star-shaped tree, an inference of the rate of evolution will depend upon specific events, but (making some assumptions) the mean inferred rate would be equal to λ. The total amount of evolution that we will have observed across our independent samples will be equal to Σ_i_ t_i_, leading to some variance in the inferred value.

Consider now the same circumstance, but with a stick-shaped phylogeny i.e. there is one underlying population which evolves at rate λ, and every sample is the consensus of that population. In this case our inferred rate of evolution will (making the same assumptions) also have mean equal to λ, but the total evolutionary time described by our samples will be only max_i_ {t_i_ }. Thus the variance in our inference will be greater.

We think we are in agreement that pseudoreplication increases the likely error in a regression-based estimate of evolutionary rate, but we are not certain that this would lead to a systematic underestimate. Possibly it does if linear regression produces a skewed estimate of rate?

We think there is a simpler explanation for at least some of the underestimate in the rate inferred by a regression method. Linear regression assumes that there is one underlying population. If there are two populations, one of which evolves faster than the other, then the assumption of there being only one population will generally fit a rate that lies between the two true rates of evolution.

Reviewer #2: 1. In Fig 3B, the regression slope looks close to 1. Might be worth adding an SI figure showing the non-synonymous rate vs the combined synonymous and non-coding rate.

This is correct. Most variants are nonsynonymous, so the nonsynonymous rate is similar to the combined nonsynonymous+synonymous rate. We have added an SI figure (S7 Figure) with the suggested data.

2. Lines 322-326: These lines should come earlier---I was struggling to understand Figure 5 without them. But I am also troubled by this assumption. I guess this is what is leading to the allele sharing. In the lineage picture, it seems much more natural to just say that multiple lineages are allowed to be present in each sample. The manuscript cites two papers in support of its "either-or" assumption. Both had the senior author of the present manuscript as an author, so I'm a bit hesitant about asserting this, but I believe that they instead show that samples tend to be "dominated" by one lineage, but with other lineages still found at low frequency. Fig 3a of the first (Kemp et al) just shows the same patient as Fgi 5 of the present manuscript, and one can see the minority lineages in the same time points. The second paper is on a quite different virus, influenza B, but still if I'm reading its Fig 1 correctly, on one of the two days (Oct 4) when clade B was observed, clade A was also observed.

We have tried to clear up the application of our results to Figure 5; hopefully this resolves some of the confusion around this point.

We have rewritten this section, noting the complexities of Figure 5:

…we assumed that each consensus sequence could be assigned uniquely to a single subpopulation. This assumption is supported by some past data describing population structure in respiratory virus infection. However, not all viruses follow this pattern, and it remains an assumption. As we note for Patient H, some minor variants seemed to be shared between samples from distinct subpopulations, reflecting that our model, which is based upon consensus sequences does not capture the full information provided by short-read data. [line 459ff.]

There are not many papers looking using genomic data to look in detail at within-host population structure during long-term respiratory infection. In the flu B case there was no substantial overlap between inferred haplotypes. We agree with the reviewer’s assessment that samples tend to be dominated by one lineage; applied to consensus sequences this implies that each sequence is representative of one of potentially multiple populations of viruses within the host.

We have provided details of the method earlier in the manuscript:

Our method fits models of different numbers of underlying populations to consensus viral sequence data from a host, assuming each sample to be representative of one of potentially multiple independent subpopulations of viruses in the host, and identifying the smallest number of subpopulations required to explain the sequence data. A rate of evolution is inferred for each subpopulation. [line 147 ff.]

3. The description of how the starting genotype is inferred when there is no sequence from the onset of infection should be clarified. Is Wuhan Hu-1 used to polarize the mutations, as in the simulations? Are subpopulations allowed to share substitutions? (Lines 420-421 seem to suggest that they can share one, but the fact that there is no ambiguity in the original genotype for >2 subpopulations suggests that they can't.)

We have added further detail about the methods. As shown in the new Figure 7, the sequence of the viral genome is effectively discarded during the processing of data; the method is interested only in the presence or absence of variation at different sites in the genome.

We have altered our method to penalise the case where sequences in different subpopulations share substitutions, but sharing one substitution is allowed. Two or more substitutions shared between sequences is not allowed.

We note that preventing the sharing of multiple substitutions is necessary for our calculation to converge in a reasonable length of time. For example, Patient H has 20 samples, such that restricting the numbers of divisions into three or four subpopulations tested is a necessity. We explored approaches to likelihood optimisation that differed from a systematic search, but we were not able to identify an effective algorithm for this. At present the three-subpopulation calculation for Patient H takes around half an hour on a high performance laptop, while the four-subpopulation calculation (necessary to validate the three-subpopulation model) takes longer than that; there is a payoff between expanding the number of options tested and the time taken to do so.

We acknowledge that we could allow more flexibility in the consensus sequence at the expense of sharing more variants between subpopulations; we decided not to implement this in the current version of our method. More details on the choice of consensus sequences is provided in S11 Figure.

4. I don't understand how not allowing subpopulations to share substitutions is a form of parsimony. Why can't one lineage acquire substitutions and then split into two that acquire different subsequent substitutions? That doesn't require any more events than a case where the splitting occurs before the substitutions.

At present our method does not consider subpopulations which split during the course of infection; the assumption is that they exist from the beginning of the infection, with evolution following a star-shaped phylogeny. Our reference for talking about parsimony is maximum parsimony phylogenetic inference, which we understand as involving the reconstruction of the evolutionary history of a population so as to include the smallest possible number of changes in genomes. In the data we are looking at we observe various changes in genome sequence; assuming that all observations of a variant arise as the consequence of a single mutation in one population reduces the number of mutations that occur on our implied evolutionary tree.

We went some way to thinking about possible splits in the underlying tree. We wrote a provisional piece of code (Model Y1) extending the IVY code to model a split of a single population into two; applying this model to the data, this model was not favoured in any of our populations. Our belief is that these kinds of split can happen but that they don’t happen in any of the cases we looked at in this manuscript.

With regard to method development, we reached the conclusion that there was more that could be done to develop IVY towards a more general piece of code to look at compartmentalisation, but that this would require significantly more work than was necessary for studying the data we present here. We therefore drew a line under the code development angle in order to write up the current piece of work. Cases in which there are splits between populations could be considered, but to do this well would need a rewrite of the code, probably from scratch.

5. The explanation of the motivation behind the dimensionality reduction approach used in Figure 1 should be clearer. What are we supposed to take away from these plots that would be obscured by, e.g., PCA? Honestly, I think Fig 1 would be stronger if it just showed the trees, with the lineage colors. The scatter plots just confused me and I didn't even notice the trees at first.

We developed the sequence maps as an alternative way of viewing the sequence data. For example, the data from Patient H show very simply that the later sequences are in three distinct regions of sequence space.

We have made some changes to Figure 1 and the general description of methods. For example, we now show multiple representations of the same data in this Figure, to provide something of an introduction to our approach.

An illustration of our method is shown in Figure 1, where we show different representations of consensus sequences collected from Patient G. [line 152ff.]

The code for generating these maps is not particularly complex. PCA is another approach to dimension reduction, but conceptually we feel it is better at describing datasets that involve high-dimensional vectors of continuous variables.

We have revised the text to de-emphasise these plots: They formed an important part of the development of our method, but do not seem to help everyone.

6. As another example of how more explanation of the biology behind the method would be helpful, I'm still not sure how to think of the epsilon parameter. Lines 313-315 say the definition "is a broad one, encompassing for example the temporary changes in consensus that might result from clonal competition". But I don't think it's ever explained what the definition actually is. And couldn't the changes in consensus sequence in Fig 5 be considered to result from clonal competition? What does the parameterization of epsilon on lines 464-466 imply about what processes are contributing to it?

We use a value for epsilon that originates from a previous study. In that study a linear regression calculation was performed, measuring the number of nucleotide changes occurring between SARS-CoV-2 sequences collected from a single host using an identical protocol for nanopore sequencing to that used to generate the sequences for the nine patients we looked at. The majority of cases studied had just two samples collected. The parameter epsilon is the value of the regression line at time t=0. The implication of the model is that consensus sequences collected from the same host on the same day have a statistical difference between them which can’t be explained in terms of evolutionary divergence. This difference is likely comprised of multiple contributing factors:

1. Simple sequencing error. For example an underlying variant, perhaps at an underlying frequency of 40%, might be observed at frequencies between 20% and 60%, depending on errors in nanopore sequencing and limited read depth. Such errors would lead to changes in the consensus sequence independent of evolution.
2. Sampling effects. For example, swabs collected from a patient might contain sets of viruses which comprised non-representative samples of an underlying population. Changes in the consensus sequence represent differences between stochastic samples.
3. Temporary changes in the sequence consensus, as might arise from clonal behaviour. Specifically, a variant within a population might rise to a frequency of greater than 50%, thus changing the consensus, before dying out, causing the consensus to revert. These changes would cause increases in the number of variants observed at any given time, independent of an underlying process of the simple gain of variants by fixation in the population over time, and leading to a linear regression model with a non-zero value at time zero.
