# Supplementary material for: Variable rates of SARS-CoV-2 evolution in chronic infections
Source: PLoS Pathog. 2025 Apr 28;21(4):e1013109. doi: 10.1371/journal.ppat.1013109 (PMC12061394; doi:10.1371/journal.ppat.1013109)
Supplement: Attachment — Submitted filename: Response_to_reviewers_auresp_2.docx [file ppat.1013109.s019.docx]

We thank the reviewers again for their consideration of our manuscript. Comments from the reviewers are included below in black text. Our responses are given in red text, with quotes from the revised manuscript in blue text.

**Editor’s comments**

Both reviewers appreciate the substantial revisions that the authors have made in response to the first set of reviews. While the first reviewer is happy with this revised manuscript, the second reviewer has several concerns that have arisen from now having a better understanding of the methodology. The concerns include whether subpopulation structure may be supported when evolutionary rate heterogeneity is the reason for observing a burst of new alleles as well as whether the ambiguous nucleotides in Patient G are meaningful (in that they might indicate something other that low sequencing quality). The reviewer also has additional major concerns. While I am recommending a minor revision, a thorough response to this reviewer's current concerns I think will require substantial thought and potentially several reanalyses, such that edits may end of being closer to a major revision.

We strongly appreciate the new comments from reviewer 2. We have made a major clarification in how we use the language of fixations and fluctuations, and have altered our method for identifying consensus sequences in two of our models, albeit that this did not change any of our results.

We have expanded our discussion of rate heterogeneity. In short, rate heterogeneity is a possibility. It is likely to have occurred in patient H due to the use of antiviral therapy in that individual. While we cannot publish details, the use of antiviral therapy was not a common event in the cohort of patients in general as, due to their occurrence early in the pandemic, antiviral therapies were few.

From a methods perspective, it would likely be difficult to build rate heterogeneity into our model given the kinds of data we are looking at. We note that, even where evolution is simulated under a constant rate, stochasticity means that the outcomes can be significantly different from one another (e.g. compare the individual inferences in S3 Figure). If it were known that a specific treatment was administered at a particular time, it would be worth considering, but this feels beyond the scope of this manuscript.

The comments on ambiguous nucleotides are of interest. We think there might be scope for a method which divides reported ‘N’ nucleotides into more than one category, albeit that the use of short-read sequence data would be of itself more efficient. Again, further work is suggested that would go beyond the scope of the present manuscript.

Reviewer 2

The authors have greatly clarified their methods and the underlying data.

Now that I understand things better, I am concerned about the level of support for the subpopulation model and the values of the evolutionary rates in patients E and F. If I understand Fig S1 correctly, in two of these cases, the evidence is simply that there was a burst of multiple substitutions in the last sample after a period of stasis in the first three samples---no parallel diversification or anything like that. I think the model favors subpopulations here because it requires substitution rates to be constant over time, while allowing them to vary across subpopulations. But in Patient H (Figure 5), it looks like in each subpopulation there is a long period of stasis followed by bursts of multiple substitutions, so I don't think this should be regarded as an unlikely scenario. To some extent, this simply means that we could re-interpret the results as being about rate heterogeneity in time rather than space, but it would make the heterogeneity even more extreme.

We previously mentioned potential effects of a non-constant rate of sequence evolution, but agree that this section is improved by a focus on patients E and F. Where there is a sudden fixation of a lot of variants, the constant-rate assumption of our model tips things in favour of two underlying populations, evolving fast and slow, respectively. A similar statement can be made about our model of fluctuations, which is also Poisson-shaped. A more heavy-tailed model of fluctuations would also weight the model towards inferring a single population.

Further to this, we discuss the use of antiviral therapies to treat SARS-CoV-2 infection. Mutagenic therapies such as molnupiravir might in particular be expected to lead to the generation of large numbers of genetic variants, and potentially fixations. We note that in Patient H the use of convalescent plasma means that a model of time-dependent rate of evolution could be supported, though the implementation of this would involve considerable complexity.

We do not have ethical consent to share all the clinical details of the patients in our study, but with samples collected from early in the pandemic, from early- to mid-2020, the use of specific antiviral drugs was sparse. Intrinsically, molnupiravir usage is not a likely hypothesis for this cohort.

We also note that there are cases where an interpretation explaining differences between samples as heterogenity in rate, rather than space, seems unlikely. In patient H we have multiple samples assigned to each of three subpopulations, differentiated by between 7 and 19 variants.

In the manuscript we have added a comparison of assumptions with linear regression: Any statistical test is conducted on the basis of assumptions, and ours is no different from this.

We have rewritten a part of the discussion to better deal with these considerations:

In common with other methods for inferring rates of evolution, our method is built around assumptions. For example, linear regression assumes that viruses in a host comprise a coherent population that evolves at a coherent rate. Our method relaxes the assumption of a single population but maintains that each population evolves at a constant underlying rate. In some cases, this assumption could affect the inference of the number of within-host populations. For example, in Patient E, sequences on days 12 and 21 equal to that on day 0 is followed by a sequence on day 31 containing five new mutations. In Patient F, something similar is observed, with sequences on days 20 and 22 differing by seven new mutations. In each of these cases, the assumption of a constant rate of evolution pushed the model towards a conclusion of emissions from differing populations, as opposed to a sudden gain of multiple variants. In particular, where antiviral drugs have the potential to accelerate evolutionary processes^37^, a model of variable evolutionary rate may be appropriate. Models of time-dependent evolutionary processes have been used to study within-host evolution^38^. However, the limited nature of the data in this case mandated a cautious approach towards more complex evolutionary models. Among the individuals in our study, Patient H was repeatedly treated with convalescent plasma^10^, suggesting that, while the division into subpopulations is strongly supported, a variable rate of evolution in each population might be considered. Within our data, conclusions in favour of distinct subpopulations are clearest where multiple samples contain shared, and distinct, variants. Inferred rates of evolution should be understood as mean values estimated over time. The timing of cases in our cohort, hospitalised in early-to-mid-2020, means that specific antiviral treatment was rare.

I am also concerned about how many ambiguous nucleotides there are in the consensus sequences at variable sites in patient G. Do the authors have any sense for what is going on here? Are these just low-quality sequences with many ambiguous sites throughout the genome, or is there something special about these sites? Perhaps polymorphism is contributing to ambiguity? Either way, it's a bit worrisome as it is, but if it's the latter and really is reflecting polymorphism, it would be a great opportunity to make the analysis much stronger.

We have looked at sequence coverage across our samples, which we had not previously considered. Patient G is distinct in having more sequences with lower coverage than other patients in the study. We have added a Supplementary Figure with this information.

The picture of ambiguous nucleotides in patient G is more complex than simply low coverage, which tends to show up in stretches within the reported sequence data. For example, position 1 in our Figure 1 is reported as an N in at least two samples where it is flanked by non-ambiguous nucleotides, not fitting the general pattern of the dataset. There might be something about this position in the genome which either a) is in some ways different from the others in terms of the sequencing process (e.g. due to RNA structure or another consideration) or b) reflects some underlying relationship between polymorphism at a site and ambiguity.

As we mention in our discussion, we believe there is potential to use short-read sequence data to gain better insights into compartmentalisation. Something towards these lines might be done using the concept that polymorphism, in some cases, leads to ambiguous nucleotide calls in consensus sequences. This would involve, in effect, converting the ambiguous N nucleotide into two, one indicating an ambiguous nucleotide arising from a lack of sequence information, and the other indicating an ambiguous nucleotide likely arising from underlying polymorphism. However, we are not sure how to achieve this in a simple manner: There would need to be some validation of any new approach to assess N nucleotides that would require the raw nanopore data used to generate the consensus sequences, and we do not have access to this.

We note that ambiguous nucleotides do not contribute towards our likelihood function: By including sequences with suboptimal coverage we are being maximally inclusive of informative data wherever we have it, without distorting our model by the inclusion of ambiguous nucleotides.

We have added a new S9 Figure showing the extent of coverage of the genome in each sequence sample.

Major comments

1. "Fixation and fluctuation sites": I'm still stuck at the point where I was before, being able to follow the algorithm as a pure algorithm (I think), but not understanding the biology. For example, suppose I have an individual sampled 6 times, and I try to write down the maximally fluctuating sequence of observations of a variant, 010101. If I'm understanding the algorithm correctly, this would be classified as a fixation, rather than a fluctuation. (The Hamming distance to both 000000 and 111111 is 3, while the distance to 011111 is 2.) The same would be true for any permutation (necessarily leaving the first 0 in place): 011100, 011010, etc. Similarly, in the real data, variant 6 in subpopulation of Patient G appears in the sequence 01010N1 and is called a fixation. What is the biological interpretation of this?

We appreciate the opportunity to explain this more clearly. We have put in some further description of fixations and fluctuations, and their role in our method. We have also clarified the concept of a fluctuation in our methodology.

Assuming a simplified model of population dynamics, we divide sites in the viral genome into two, namely those at which a permanent change in the consensus genome sequence occurs, and those at which no such event occurs. Data in viral sequences is then treated as noisy observations of the underlying consensus. Observed sequence variation reflects either these ‘fixation’ events, or ‘fluctuations’, namely deviations from the true underlying state of the population. Our model fits a constant rate of evolution to a population, reflecting the number of fixation events: More fixation events in a given time implies a faster rate of evolution. By contrast, excessive fluctuation events are penalised. Biologically speaking, allele frequencies may fluctuate either side of the consensus cutoff, and genome sequencing may generate errors in the consensus, but these events occur at a limited rate. Allowing for more than one population in a host may allow fewer observed variants to be interpreted as fluctuations: Where this causes a significant improvement in model likelihood, the more complex population structure is inferred.

… identifying ‘fixation’ and ‘fluctuation’ sites for each subpopulation (Figure 7D), representing sites in the genome at which variation was observed, for which the modelled underlying consensus sequence either did or did not undergo a permanent change.

We have subtly changed the language of part of our methods section to no longer speak about fluctuation sites: This was confusing.

In our thinking, we have an idealised model of changes at positions in the genome, and then sequence data that is emitted from that position. In the idealised model only one of two things can happen: Either there is, at some point, a permanent change in the consensus sequence, such as 00011111, or there is no change in the consensus sequence, i.e. 0000000. The former set of sites are referred to as fixation sites, but no name is now given to the latter.

At fixation sites, the time of fixation is defined by the first occasion on which a change in consensus is observed. This has consequences to which we return later.

Now, any observation of a variant in a sequence falls into one of two categories. Either it reflects an underlying fixation, or it is a ‘fluctuation’. Fluctuations include the observation of a 0 after a fixation event e.g. in the sequence 011011, the latter zero is such.

Sites which provide a strong mismatch to our idealised sites underpin our inference of within-host structure. For example, a variant that was 010101 could have a variety of interpretations. Biologically it could represent a site which, potentially via a combination of selective effects at linked sites, undergoes a series of changes in allele frequency which take it back and forth across the consensus boundary. Alternatively, it could be a site at which the true consensus shifts from 0 to 1, but in which there are a series of errors in the sequencing process, perhaps if the variant frequency remains within a window close to 50%. Alternatively, it could represent alternating observations of two within-host populations, one of which has a rapid fixation event at that site, while the other has no fixation event: Adding an additional 0 representing the consensus sequence prior to the first sample, the populations would be 0111 and 0000 over time. We note that the hypothesis in this case of a single population requires two fluctuations at the site, with 011111 the best idealised match to the observation. However, the hypothesis of two split populations requires no fluctuations: This is the root of our inference method.

The sequence 01010N1 is functionally equivalent to the sequence 010101. The N in this case represents missing data, so is not counted in any way when assigning the variant to be either a fixation or fluctuation.

2. I still don't totally understand how the initial genotype is inferred. In Model X, if there is a polymorphism in which two subpopulations have one allele and two subpopulations have the other, how is it determined what the initial sequence was? In both Model X and Model Y, what happens when sites are polymorphic within a subpopulation and both alleles appear in other subpopulations?

We have made a change to our method for Models Y and X which in some ways parallels the approach taken in Model V.

In Model V, we have two first samples, one from each hypothesised subpopulation. If these contain variants that are distinct from one another, we hypothesise that the initial consensus must be something ‘in between’ the two sequences, but don’t know what it is. We loop over the various combinations of variants to infer the best initial consensus.

In Models Y and X, if a variant is distinct to one of the three or four first samples, we hypothesise that it arose in the respective subpopulation. In the case that a variant is shared (e.g. we have {0, 1, 1} nucleotides for the first three samples in Model Y), we use the same algorithm as for Model V, but considering potential nucleotides at shared variant sites, rather than distinct variant sites.

Our alteration to the code is described as follows:

Models Y and X describe the evolution of three and four populations respectively, with a common initial consensus. In constructing a consensus, where the first sample from only one population described a variant, that variant was assumed to have arisen in that population. However, sites for which the first samples from more than one population contain a variant are ambiguous. An ensemble of models were generated in which the consensus sequence was one of the set of possible sequences with a 0 at every site for which a variant was seen in the first samples from zero or one populations, and either a 0 or a 1 at every site for which a variant was seen in the first samples from two or more populations. The maximum likelihood was then calculated, considering all possible consensus sequences in turn.

Our reason for not having done this previously is that shared polymorphism in more than one population is something we assumed to be intrinsically unlikely. Variants are not expected to appear independently more than once, in separate populations, over the course of infection. As such, a small penalty is ascribed to this event happening once, while any division of samples whereby sequences in different populations share two or more variants is prohibited.

Rerunning our method on the patient data did not produce any change in the likelihoods or parameter estimates generated by our code.

Minor revisions

1. Having the consensus sequence represent a single subpopulation is a very mild assumption. Even without any subpopulation structure, you'd expect that typically the consensus sequence would reflect a single lineage rather than a chimera. I think the authors can be pretty confident here. Maybe Figure 5 could be redone to show how the consensus sequence is always from just one lineage even when other lineages are detected?

We aren’t entirely clear what the reviewer is asking for in this case. Every observed sequence is assigned to a subpopulation, but the consensus sequence can be different to all of the observed sequences. For example, all of the samples may contain variants, when at the same time the consensus does not contain any variants. We have clarified the legend of Figure 5 to state that the consensus sequence is included in all of the plots.

2. Line 566, "the assumption that variants may be gained but never lost": I am not sure what this phrase means---columns do go from 1 to 0 within a subpopulation in the data.)

We have substantially rewritten this section of the text, removing the phrase in question.

3. Lines 167-169: "We note that the variants C5T and C7T could not be distinguished by our method: Under our maximum likelihood reconstruction one was inferred to be a fluctuation event, with the other being a fixation.": I would take another sentence or two to clarify this.

We have added details for clarification.

In our analysis no distinction could be made between the variants C5T and C7T. Where the first observed case of a variant is in the last collected sequence, the nature of this change is not easily recognisable. Under our maximum likelihood reconstruction one of C5T or C7T was inferred to be a fluctuation, with the other being a fixation, but with no ability to assign which was which
